# Simulation of Stress Field during the Selective Laser Melting Process of the Nickel-Based Superalloy, GH4169

**DOI:** 10.3390/ma11091525

**Published:** 2018-08-24

**Authors:** Zhanyong Zhao, Liang Li, Le Tan, Peikang Bai, Jing Li, Liyun Wu, Haihong Liao, Yahui Cheng

**Affiliations:** School of Materials Science and Engineering, North University of China, Taiyuan 030051, China; syuzzy@126.com (Z.Z.); nucliliang@126.com (L.L.); hltanle@foxmail.com (L.T.); jing.li3d@hotmail.com (J.L.); wuliyunnuc@126.com (L.W.); scizhao@foxmail.com (H.L.); nuccyh@foxmail.com (Y.C.)

**Keywords:** selective laser melting, GH4169, temperature and stress fields, simulation, model

## Abstract

In this paper, GH4169 alloy’s distributions of temperature and stress during the selective laser melting (SLM) process were studied. The SLM process is a dynamic process of rapid melting and solidification, and we found there were larger temperature gradients near the turning of scan direction and at the overlap of the scanning line, which produced thermal strain and stress concentration and gave rise to warping deformations. The stresses increased as the distance became further away from the melt pool. There was tensile stress in the most-forming zones, but compressive stress occurred near the melt pool area. When the parts were cooled to room temperature after the SLM process, tensile stress was concentrated around the parts’ boundaries. Residual stress along the z direction caused the warping deformations, and although there was tensile stress in the parts’ surfaces, but there was compressive stress near the substrate.

## 1. Introduction

As a precipitation aging-enhanced nickel-based superalloy, GH4169 alloys are used extensively in the important high-temperature parts of the aerospace and nuclear industry due to its good corrosion resistance, anti-radiation, and excellent mechanical properties. Traditional manufacturing methods of GH4169 nickel-based superalloy may not have been the best choice, due to processing problems such as tool consumption, processing complex, high costs, and complex processes. Maybe this is why the application of selective laser melting (SLM) technology to form GH4169 nickel-based superalloy has caused widespread interest, as it is low-cost, has a simple process, and uses direct molding parts [1].

SLM technology can fabricate metal parts with complex shape, good mechanical properties, high precision, and high density [2], which cannot be produced using traditional methods [3,4]. SLM has been widely used in the fields of medical, military, aerospace, and automobile manufacturing because it is now able to process a variety of metals, including, but not limited to, chemical elements such as Al, Cu, Ti, etc. [5,6,7]. Up till now, many researchers have also carried out research on this technique: Michael et al. investigated the SLM process of the nickel-based superalloy IN738LC and the cobalt based alloy Mar-M509, where results showed that the microstructural and mechanical characteristics were attributed to the recovery and recrystallization behavior of IN738LC and Mar-M509 [8]; Vilaro et al. was able to prepare the Nimonic 263 with good microstructure and mechanical properties by using SLM [9]; Xia et al. established the mesoscopic model to investigate the thermodynamic mechanisms and densification behavior of nickel-based superalloy during additive manufacturing/three-dimensional (3D) printing (AM/3DP) processes [10]; Pröbstle et al. investigated creep properties of the polycrystalline nickel-based superalloy prepared by SLM, and found that the parts prepared by SLM had better creep strength than that of conventional casted parts [11]; Fabian et al. studied correlating laser-scanning strategies with the resulting textures and corresponding anisotropy of the elastic behavior of bulk materials [12]; and Carter et al. developed a processing route for the SLM powder-bed fabrication of the nickel superalloy CM247LC, and found that the island scan strategy strongly influenced the grain structure of the material [13].

The SLM process is a dynamic one of rapid melting and solidification with severe temperature gradients producing large thermal strain and stress concentration, resulting in warping deformations and cracks in the parts [14]. The distributions of temperature and stress in the alloy were difficult to measure by using the traditional method, prompting the widespread use of the finite element simulation method instead for the analysis of the temperature and stress field distributions [15,16]. Gu et al. established a three-dimensional, transient, finite element method (FEM) model to predict the stress distribution of parts shaped during the SLM process. By simulating the laser-beam scanning process, the peak values of the thermal stresses were first recorded at the onset of the first track where the first heating-cooling cycle occurred. After the whole part was cooled down, the largest residual stresses were found at the end of the first and last tracks. The simulation results were then verified by conducting the experimental investigation with the same parameters [17]. Hodge et al. discussed various perturbations of the process parameters and modeling strategies and compared the model-generated solid mechanics results [18]. Hussein et al. used three-dimensional finite element simulation to investigate the temperature and stress fields in single 316L stainless steel layers built on the powder bed without support in SLM [19]. Wu et al. established proper numerical models to investigate the residual stress evolution of AlSi10Mg alloy in a point exposure SLM process [20].

Although finite element simulation for the temperature and stress of the parts prepared by SLM has been carried out, due to differences in the physical properties of the materials, the distributions in temperature and stress of GH4169 alloys during the SLM process are difficult to predict. Furthermore, residual stress distribution in the parts prepared by SLM, which cool to room temperature, needs further research as temperature distribution and stress fields of GH4169 alloys’ parts during the SLM process are the main subjects of study. Our aim in this study was to disclose the distributions of temperature and stress during the SLM process by establishing proper three-dimensional finite element models and providing theoretical guidance for the SLM formation of GH4169 alloys.

## 2. Experiment and Simulation

### 2.1. Experiment

The experimental equipment used was the Nd:YAG laser (LWY400P, Huagong Ltd., Wuhan, China), and the substrate plate was Q235. The experimental material used was GH4169 alloy powder, where its size range was 20–50 μm. The effects of the scanning process on distributions of temperature and stress during the SLM process were studied.

### 2.2. The Calculation Model

#### 2.2.1. Finite Element Method for Transient Heat Conduction

The SLM is a rapid and intense forming process that uses the interaction of a laser beam. The material undergoes complex processes, such as thermal conduction, heat loss due to convection and radiation, phase transformation, and melting and cooling solidification. Therefore, a three-dimensional transient temperature field model was first established. During the heat transfer process, we assumed that the powder bed and its surroundings would constitute a closed and thermally insulated system. Energy balance follows the first law of thermodynamics, and in the Cartesian coordinate system, the temperature field of *Ω* can be expressed as a three-dimensional heat transfer differential equation, such as the following [21]:

(1) $$\rho c\frac{\partial T}{\partial t}=Q+k_{x}\frac{\partial^{2}T}{\partial x^{2}}+k_{y}\frac{\partial^{2}T}{\partial y^{2}}+k_{z}\frac{\partial^{2}T}{\partial z^{2}}\;\;\left(x,y,z\in \Omega \right)$$

where ρ is material density (kg/m^3^); *c* is the specific heat capacity (J/(kg·°C)); *T* is temperature (°C); *t* is interaction time (s); *k_x_, k_y_, k_z_* are the effective thermal conductivity of the powder bed in the x, y, and z directions, respectively (W/(m·K)); and *Q* is the heat generated per volume within the component (W/m^3^), which is described more specifically in the following sections.

In order to solve the heat transfer differential equation, the initial and boundary conditions need to be determined. Before the forming process begins, we assumed that the initial temperature was *T_0_*, which can be expressed by:

(2) $$T\left(x,y,z,0\right)=T_{0}\;\;\left(x,y,z\in S\right)$$

In the SLM process, the surface of the powder bed interacting as a laser heat source is simplified to the heat flux input, which belongs to the second type of boundary conditions. This can be defined by:

(3) $$k_{x}\frac{\partial T}{\partial x}n_{x}+k_{y}\frac{\partial T}{\partial y}n_{y}+k_{z}\frac{\partial T}{\partial z}n_{z}=Q$$

The convective heat dissipation process with air (or protective atmosphere) as the medium on the surface of the powder bed belongs to the third type of boundary condition, which can be defined by:

(4) $$k_{x}\frac{\partial T}{\partial x}n_{x}+k_{y}\frac{\partial T}{\partial y}n_{y}+k_{z}\frac{\partial T}{\partial z}n_{z}=h\left(T-T_{0}\right)$$

The surface of the powder bed radiates thermal energy to the surrounding environment and belongs to the fourth type of boundary condition, which can be defined by:

(5) $$k_{x}\frac{\partial T}{\partial x}n_{x}+k_{y}\frac{\partial T}{\partial y}n_{y}+k_{z}\frac{\partial T}{\partial z}n_{z}=\sigma \varepsilon \left(T^{4}-T_{0}^{4}\right)$$

In summary, the temperature field boundary condition can be defined as:

(6) $$k\frac{\partial T}{\partial n}=Q+h\left(T-T_{0}\right)+\sigma \varepsilon \left(T^{4}-T_{0}^{4}\right)\;\;\left(x,y,z\in S\right)$$

In Equations (3)–(6), *n* is the normal vector of the top surface S; *h* is the heat transfer coefficient of the natural thermal convection; *T*_0_ is the ambient temperature, considered to be 25 °C; σ is the emissivity; and ε is the Stefan-Boltzmann constant, which has the value of 5.67 × 10^8^ W/m^2^ K^4^.

#### 2.2.2. Basic Theory of Stress Field Simulation

The stress perpendicular to the scanning surface is called the normal stress σ, and the normal stress along the x, y, and z axes are expressed as σ_x_, σ_y_ and σ_z_. Stress tangent to the scanning surface is called shear stress, and the shear stress along the x, y, and z axes are expressed as τ_x_, τ_y_, and τ_z_. As there will be some residual stress formed after the cooling solidification stage, if the residual stress was greater than the yield strength of the material, it will produce local deformation. Thus, the equivalent stress of Mises is the stress at yield [22].

During the SLM process, the material strain rate $\dot{\varepsilon }$ influenced by external force and temperature includes the elastic strain rate ${\dot{\varepsilon }}^{e}$, plastic strain rate ${\dot{\varepsilon }}^{p}$, creep strain rate ${\dot{\varepsilon }}^{c}$, and the strain rate caused by temperature change ${\dot{\varepsilon }}^{T}$. Their relationship can be described as [23]:

(7) $$\dot{\varepsilon }={\dot{\varepsilon }}^{e}+{\dot{\varepsilon }}^{p}+{\dot{\varepsilon }}^{c}+{\dot{\varepsilon }}^{T}.$$

The elastic constant varying with temperature can be defined by:

(8) $${\dot{\varepsilon }}^{e}=\frac{d\left(D_{e}^{-1}\sigma \right)}{dt}=D_{e}^{-1}\dot{\sigma +\frac{d}{dt}}\left(D_{e}^{-1}\right)\sigma$$

where $D_{e}$ is the elastic matrix, which is the time derivative of stress.

Based on the flow theory, plastic strain rate can be determined by:

(9) $${\dot{\varepsilon }}^{p}=\dot{\lambda }\frac{\partial F}{\partial \sigma }$$

where *F* is the yield function, $\dot{\lambda }$ is the plastic growth factor.

Based on the creep theory, the creep strain rate can be described as:

(10) $${\dot{\varepsilon }}^{c}=\frac{3}{2}\frac{{\bar{\varepsilon }}^{c}}{\bar{\sigma }}\sigma^{\prime }$$

where $\bar{\sigma }$ is equivalent stress, ${\bar{\varepsilon }}^{c}$ is equivalent creep strain rate, and ${\bar{\varepsilon }}^{c}$ can be described as:

(11) $${\bar{\varepsilon }}^{c}=\frac{d{\bar{\varepsilon }}^{c}}{dt}=\frac{\sqrt{2}}{3}{\left[{\left({\dot{\varepsilon }}_{11}^{c}-{\dot{\varepsilon }}_{22}^{c}\right)}^{2}+{\left({\dot{\varepsilon }}_{22}^{c}-{\dot{\varepsilon }}_{23}^{c}\right)}^{2}+{\left({\dot{\varepsilon }}_{33}^{c}-{\dot{\varepsilon }}_{11}^{c}\right)}^{2}+6{\left({\dot{\varepsilon }}_{12}^{c}{}^{2}+{\dot{\varepsilon }}_{23}^{c}{}^{2}+{\dot{\varepsilon }}_{31}^{c}{}^{2}\right)}^{2}\right]}^{\frac{1}{2}}$$

The temperature strain rate can be defined by:

(12) $${\dot{\varepsilon }}^{T}=\dot{T}A_{1}$$

where *A*_1_ can be described by ${Α}_{1}=a{\left\{1,1,1,0,0,0\right\}}^{T}$, *a* is the linear expansion coefficient, and $\dot{T}$ is the change rate of temperature with time.

Therefore,

(13) $$\dot{\varepsilon }=D_{e}^{-1}\dot{\sigma }+\left(\frac{d}{dt}D_{e}^{-1}\right)\sigma +\dot{\lambda }\frac{\partial F}{\partial \sigma }+\;{\dot{\varepsilon }}^{c}+\;{\dot{\varepsilon }}^{T}$$

The type (14) multiplied by the elastic coefficient matrix can be calculated as:

(14) $$\dot{\sigma }=D_{e}\dot{\varepsilon }-\dot{\lambda D_{e}}\frac{\partial F}{\partial \sigma }-D_{e}^{-1}\left({\dot{\varepsilon }}^{c}+{\dot{\varepsilon }}^{T}\right)+\frac{dD_{e}}{dt}\;{\dot{\varepsilon }}^{c}$$

When the yield condition is:

(15) $$F\left(\sigma_{ij},\;\varepsilon_{ij}^{p},T\right)=0$$

It can be calculated:

(16) $$\dot{\lambda }=\frac{q^{T}D_{e}\dot{\varepsilon }-q^{T}D_{e}\left({\dot{\varepsilon }}^{c}+{\dot{\varepsilon }}^{T}\right)+q^{T}\frac{dD_{e}}{dt}{\dot{\varepsilon }}^{c}+\frac{\partial F}{\partial T}\dot{T}}{p^{T}q+q^{T}D_{e}q}$$

(17) $$q=\frac{\partial F}{\partial \sigma }$$

Where *p^T^* and *q^T^* are the corresponding matrices, the incremental elastic–plastic strain can be described as [24].

(18) $$\sigma =\left[D_{e}-\frac{D_{e}q{\left(D_{e}q\right)}^{T}}{W}\right]\left(\dot{\varepsilon }-{\dot{\varepsilon }}^{c}-{\dot{\varepsilon }}^{T}+{\dot{\varepsilon }}^{e}\right)-D_{e}\frac{\partial F\dot{T}}{\partial TW}$$

(19) $$W=p^{T}q+q^{T}D_{e}q$$

#### 2.2.3. Mechanical Properties of Materials

GH4169 was used as the melting material, and its melting temperature is 1260–1320 °C. The mechanical parameters and coefficient of the thermal expansion of GH4169 alloys are listed in Table 1 and Table 2. The process parameters in the simulation and experiments are shown in Table 3, which, through the experiments and simulations, have been proven to result in high-quality products.

#### 2.2.4. The Moving Heat Source Model

The accuracy of the temperature and stress field simulations were affected by the heat source model. LWY400P-type YAG-pulsed laser equipment was used, and its laser heat source was loaded into the powder bed in the form of a heat flux, which obeyed the Gauss distribution [25]:

(20) $$Q=\frac{2AP}{\pi \omega^{2}}exp\left(-\frac{2r^{2}}{\omega^{2}}\right)$$

where *q* is laser power density, *P* is laser power, and *A_2_* is the heat absorption rate of materials. In the study, *A*_2_ was optimized as 0.38, the wavelength of the laser beam was 1.06 μm, *ω* is the laser spot radius, and *r* is the distance from a point on the surface of the powder bed to the center of the laser beam. This can be determined by:

(21) $$r={\left(x-x_{0}\right)}^{2}+{\left(y-y_{0}\right)}^{2}$$

**Figure 1:**
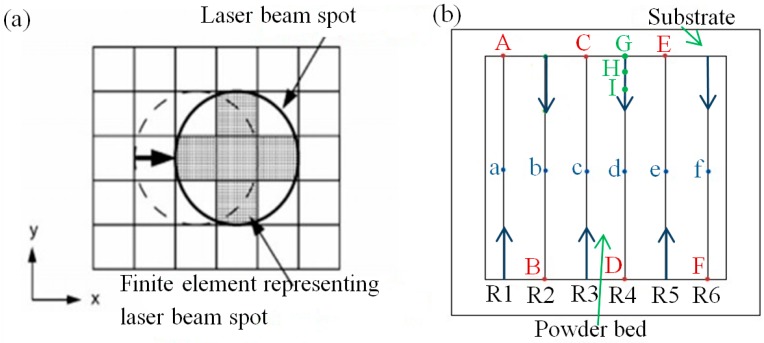
Movement of the laser beam spot and schematic diagram of powder bed. (**a**) Movement of laser beam spot by five elements; (**b**) Scanning strategy.

In Figure 1a, a simplified model of the Gauss heat source is described. The scope of the laser beam was approximated as being circular, which accounted for a cell size of 3 × 3. The heat flow density in the middle shaded area was 1, and in the four blank corners is 0.5. The scanning strategy is shown in Figure 1b [21].

**Figure 2:**
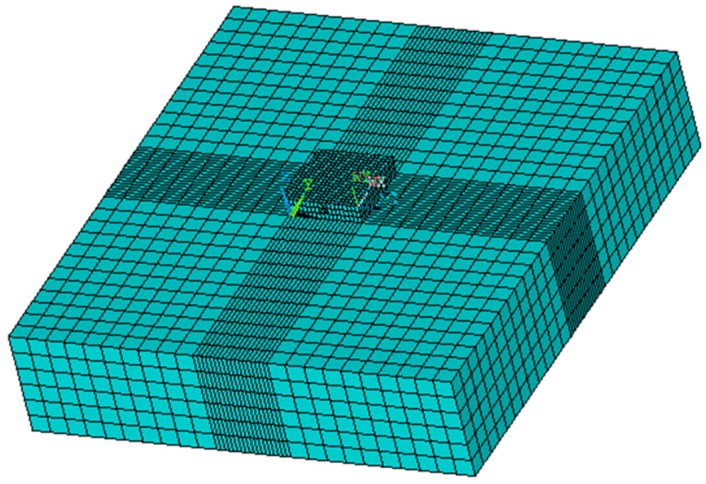
The single-layer finite element model.

The three-dimensional finite element model was divided into two components: powder bed and substrate. The size of the three-layer model was 0.6 mm × 0.6 mm × 0.45 mm. An eight-node hexahedron SOLID70 3D solid element was adopted as the mesh type in the model, and its dimension was 0.033 mm × 0.033 mm × 0.033 mm. Meanwhile, the mesh size of Q235 was 4 mm × 4 mm × 0.9 mm, and a three-dimensional, twenty-node thermal solid element, SOLID90, was adopted as the mesh type. The effect of the substrate temperature on the powder bed could be minimized by using the partition of different units with the powder bed mode. The PCG solver was mainly used for this simulation and the birth–death element method was used to load the heat source onto different units at different times. The finite element model is shown in Figure 2.

## 3. Results and Discussions

### 3.1. Temperature Distribution during the SLM Process

**Figure 3:**
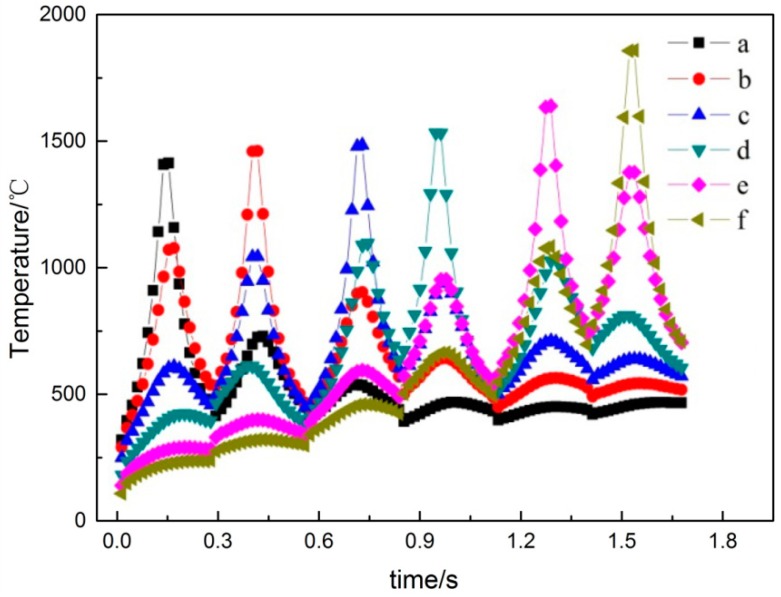
The temperature curves of points a, b, c, d, e, and f.

**Figure 4:**
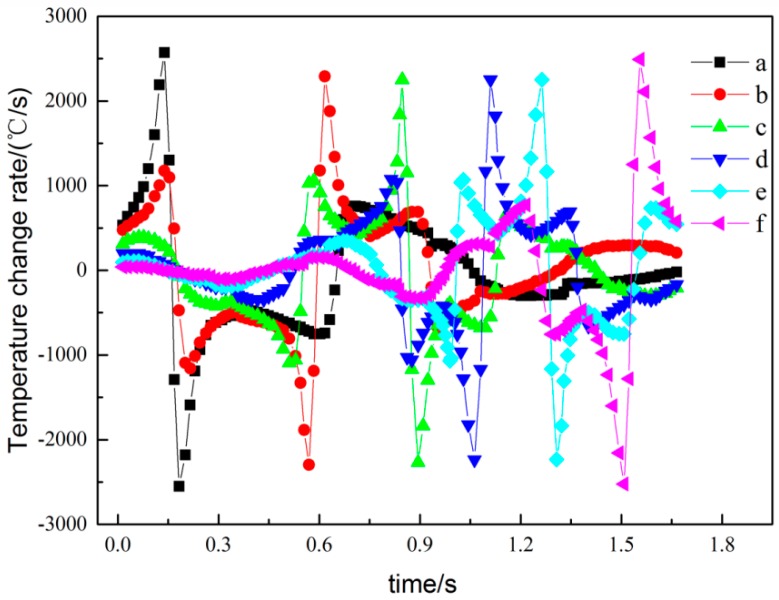
The temperature change rate curves of points a, b, c, d, e, and f.

Figure 3 shows the temperature curves of the points a, b, c, d, e, and f in Figure 1, with the changes in interaction times. Figure 4 is the corresponding node temperature change-rate curve. We will be discussing the two curves by taking point a as an example, where the curves of the other points can then be deduced by analogy.

When the laser beam closes into midpoint a of the scan line, the temperature gradually increases while the heating rate increases rapidly. The maximum values of the temperature and heating rates can be obtained when the laser beam reaches the midpoint, as shown by t = 0.17 s in Figure 3 and Figure 4, respectively. When the laser beam leaves the point, the temperature of point *a* begins to decrease, while the cooling rate begins to increase. The laser beam continues to scan toward point *b* for point *a*, during which the laser beam separation process and, afterwards, the laser beam approach is experienced. Accordingly, the temperature at point *a* decreases at first and then rises slowly. As shown in Figure 3, the time at which the second peak of point *a* and the highest temperature of point *b* occur coincide exactly.

Based on the above discussion, the temperature change trend of each scan line has six peaks, and the curve change trend is basically the same. The maximum temperature was obtained when the laser beam scanned the point. When the overlapping area between two adjacent tracks was formed, the temperature increased; however, the heat effect of the other scanning line on the point was less. As shown in Figure 3, because of the heat accumulation effect, the maximum temperature from point *a* to point *f* slightly increased. The first formed area has a preheating effect on the post-forming area, and the post-formed area re-melts the previously formed area to achieve metallurgical bonding, thereby ensuring the quality of the formed part. The heating rate was described as being greater than zero, and the cooling rate was described as being less than zero, as shown in Figure 4. The SLM forming process had high heating and cooling rates, thus causing higher temperature gradients and related thermal stresses in the parts.

**Figure 5:**
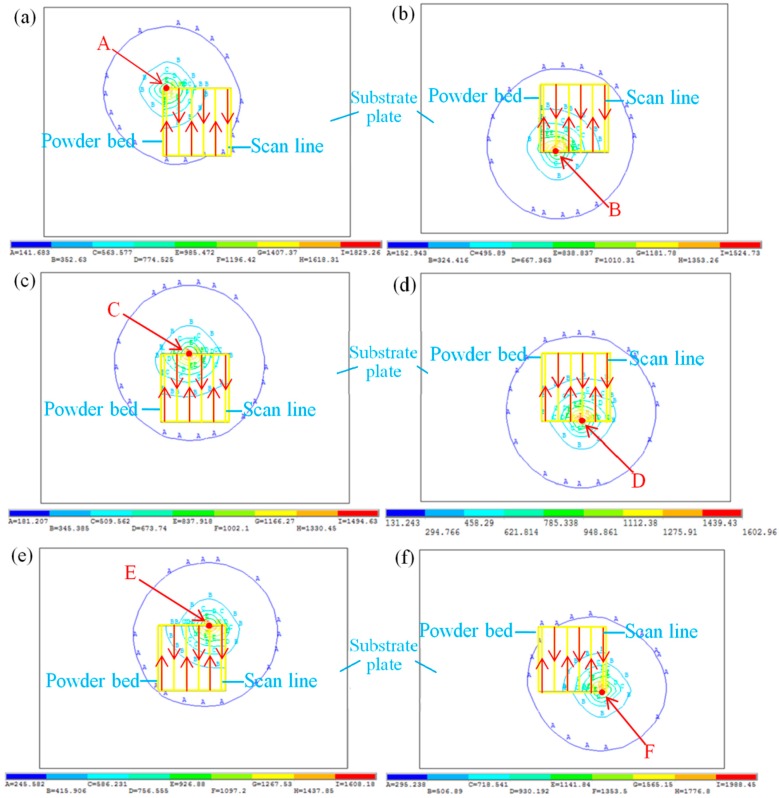
The isothermal diagrams of the endpoint of the scan line. (**a**) Point A; (**b**) Point B; (**c**) Point C; (**d**) Point D; (**e**) Point E; (**f**) Point F.

Figure 5 shows the isothermal diagrams at the endpoint of each scan line in the single layer scanning. There was a certain preheating function in the vicinity of the powder bed under the action of the laser heat source. The densest temperature isotherm around the laser heat sources caused the steep temperature gradient. Simulation results show that the maximum temperatures from point A to point F at the end of each scan line are 1829, 1524, 1494, 1602, 1708, and 1988 °C, respectively, which is different to the temperature change of the midpoint of the adjacent scanning line. It is shown that at first, the temperature decreased due to the good absorption of powders to laser radiation. Afterwards, the cumulative heat of the powder bed made the temperature increase. The temperature reached a maximum point at the end of the scan line, which had a greater effect at the beginning of the next scan line due to the complexities in thermal convection and heat radiation. It can thus be said that it is easy to cause thermal stresses and deformations with the turning of scan direction.

### 3.2. Stress Analysis

#### 3.2.1. Stress Field Distribution during the SLM Process

**Figure 6:**
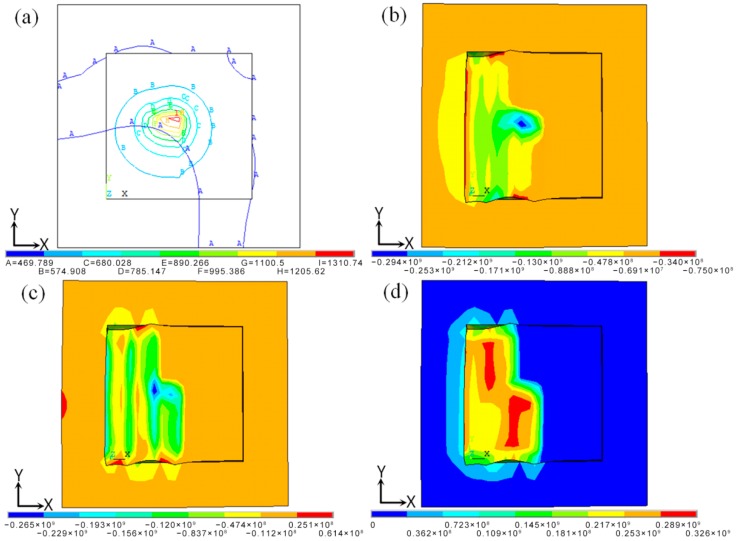
The isotherm temperature and stress fields during the selective laser melting (SLM) process. (**a**) The isotherm temperature field; (**b**) Tensile stress distribution; (**c**) Compressive stress distribution; (**d**) Equivalent stress distribution.

The isothermal temperature and stress fields during the SLM process are shown in Figure 6. In the center of the laser heat source, the temperature gradient was larger (Figure 6a), which resulted in compressive stress. In order to maintain the balance, the low-temperature region also had a corresponding tensile force (Figure 6b), and the large tensile stress caused warping deformations or cracks at the boundary of the forming part. As shown in Figure 6c, whereas the alloy powder’s lower-temperature zone created obvious tensile stress, compressive stress was formed in the higher-temperature zone, although the stress concentration and stress value were small. Stress was formed in the forming part due to the temperature gradient, resulting in the deformations. As shown in Figure 6d, the Von Mises stress equivalent was concentrated at the overlap of the scanning line, where the stress value was 326 MPa, which closed to the yield strength of the material. That was because the temperature variation of the SLM process was complex and severe, as shown in Figure 3 and Figure 4. When the adjacent scanning lines overlapped, the temperature difference between the melting liquid metal and solidification parts was bigger, and thus caused higher temperature gradients, leading to instantaneous stress close to or exceeding the yield strength, and thus easily causing cracks and deformations.

**Figure 7:**
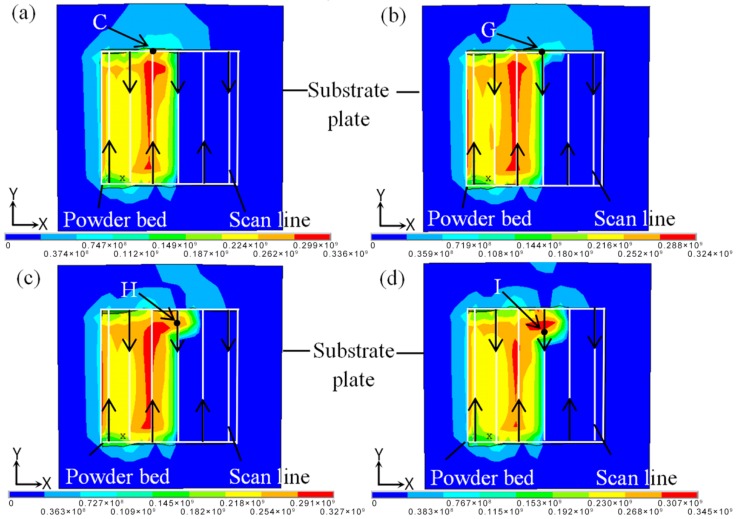
The stress distribution fields near the turning of scan direction. (**a**) Point C; (**b**) Point G; (**c**) Point H; (**d**) Point I.

As shown in Figure 3, Figure 4 and Figure 5, thermal stress and deformation occurred easily due to complex temperature variation near the turning of scan direction. The corresponding stress field is shown in Figure 7. There was a large amount of stress concentration at point C, while the stress value decreased when the laser beam scanned point G—however, the stress concentration did not decrease due to the large temperature gradient. The range of stress concentration was greatly reduced when the laser beam moved to point I, reason being because the appropriate forming lap ratio made a good lap between adjacent scan lines and thus caused re-melting, which made the residual stress in the interior release and the stress concentration reduce.

#### 3.2.2. Residual Stress Distribution of the Product at Room Temperature

**Figure 8:**
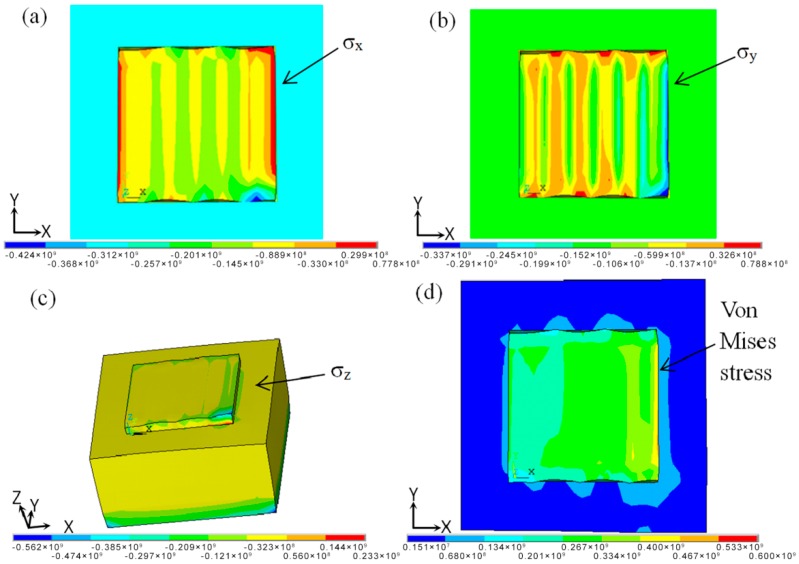
The residual stress fields cooling to room temperature. (**a**) Residual stress σ_x_ distribution; (**b**) Residual stress σ_y_ distribution; (**c**) Residual stress σ_z_ distribution; (**d**) Equivalent stress distribution.

Figure 8 shows the residual stress filed when the parts cooled to room temperature. The residual stress σ_x_ of the forming area was mainly tensile stress (Figure 8a). The maximum value of tensile stress was obtained near the boundary of the parts, which was because the boundary region was not hindered by the surrounding powder. This resulted in greater tensile stress during the thermal expansion process of the materials, ultimately causing cracks and warping deformations. Figure 8b shows that larger tensile stress was the key component of residual stress σ_y_, which occurred in the turning of scan direction. The compressive stress was concentrated in the overlapping area of the scanning line, which was because the scanning direction of single-layer scanning was along the y axis, and changed direction at the x axis. The longer cooling time resulted in a lower temperature gradient and stress concentration than when it had a longer scanning distance along the y axis. In the scan direction turning process, the short scanning distance and large temperature gradient, was what caused stress concentration. The residual stress σ_y_ was the main reason for the formation of cracks and deformations in the turning of scan direction. The residual stress σ_z_ along the *z* direction was the main cause of warpage, as shown in Figure 8c. This is because the directions of the compressive stress caused by the lower temperature in the substrate and the tensile stress caused by thermal expansion in the powder bed were contrary to one another, making it more likely to cause warping deformations. This defect could be reduced by preheating the substrate. The value of the equivalent stress of Von Mises did not exceed the material yield strength, which could ensure the quality of the forming parts (Figure 8d).

**Figure 9:**
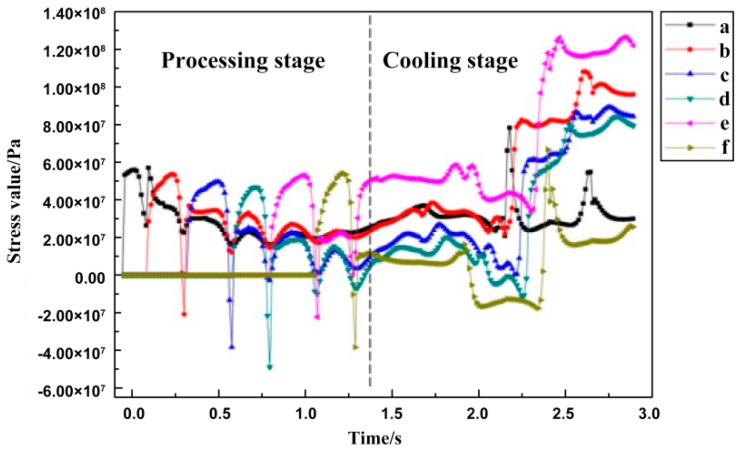
The variation of residual stress σ_x_ with time at the midpoint of each scan line.

In order to observe the change in internal stress during the SLM process, the curve of the residual stress σ_x_ at the midpoint of each scan line was plotted and the change trend of each point was roughly the same, as shown in Figure 9. The corresponding temperature distributions and temperature change rates of the midpoints of each scan line are shown in Figure 3 and Figure 4. When laser beam scanning reached the midpoint, the value of residual stress σ_x_ decreased accordingly due to the heat concentration and rise in temperature. As the laser beam moved away, the value of residual stress σ_x_ increased accordingly. During the cooling process after processing was completed, the overall trend in the changes in residual stress σ_x_ was generally stable at the beginning, and then increased rapidly. This occurred because there was a certain temperature when the forming process had just finished. In order to balance out the temperature gradient, the internal stress of the forming parts changed gradually and finally became stable after the cooling of the forming parts, which formed the residual stress σ_x_.

**Figure 10:**
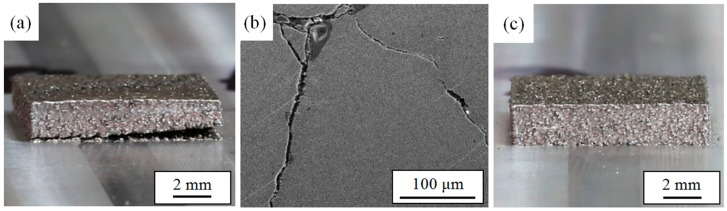
Experimental SLM-forming samples. (**a**) Warping deformations in the sample; (**b**) Cracks in the sample; (**c**) A high-quality sample.

Warping and cracking in the formed parts have always been a problem in the SLM-forming process. The most fundamental reason for this phenomenon is that the interaction with the laser heat source unbalances the powder bed and forms a steep temperature gradient, resulting in inconsistent shrinkage of the material system. Figure 10a shows a typical warping deformation sample. It is clear that there is obvious crack at the bottom of the formed part, and the experimental results are consistent with the simulation analysis. During the molding process, as the scanning area and layer thickness increase, warping first occurs at the boundary region of a single layer caused by the inappropriate process parameters. Contact with the simulation analysis knows that because of the excessive stress inσ_x_, σ_y_ and σ_z_ at the junction of the formed part and the substrate, cracks and warpage occur. Under the guidance of the simulated results, we were able to obtain high-quality SLM parts (Figure 10c) using optimized process parameters. Furthermore, common methods for reducing defects include preheating the substrate and using it while having the thermal expansion coefficient slightly larger than the SLM-formed part, which can effectively reduce the temperature gradient.

## 4. Conclusions

(1) The SLM process was a dynamic process of rapid melting and solidification, and there were larger temperature gradients near the scan direction turning point and at the overlap of the scanning line, which produced thermal strain and concentrations of stress, and also gave rise to warping deformations.

(2) The stresses increased as the distance away from the melt pool also increased. There was tensile stress in zones with the highest levels of concentration, but compressive stress occurred near the melt pool area. 

(3) After the SLM process, when the parts were cooled to room temperature, the tensile stress concentrated on the boundary of the parts. Residual stress along the z direction caused the warping deformations. Although there was tensile stress in the parts’ surfaces, there was compressive stress near the substrate.

## Figures and Tables

**Table 1:** Mechanical properties of GH4169 alloy.

Temperature/°C	20	100	200	300	400	500	600	700
Elastic Modulus/GPa	205	201	196	189	183	176	169	164
Shear Modulus/GPa	79	77	70	73	70	67	64	61
Poisson Ratio	0.3	0.3	0.3	0.3	0.31	0.31	0.32	0.34

**Table 2:** Coefficient of thermal expansion of GH4169 alloy.

Temperature/°C	100	200	300	400	500	600	700	800	900	1000
Thermal Expansion Coefficient/10^−6^·°C^−1^	13.2	13.3	13.8	14	14.6	15	15.8	17	18.4	18.7

**Table 3:** The process parameters in the simulation and experiments.

Parameter	100
Laser Power	150 W
Scanning Speed	150 mm/min
Hatching Space	100 μm
Spot Diameter	150 μm
Layer Thickness	50 μm
